# A Delicate Balance: Challenges in the Management of Primary Hyperparathyroidism and Congestive Heart Failure

**DOI:** 10.7759/cureus.39854

**Published:** 2023-06-02

**Authors:** Nikita Mohan, Rupinder K Bahniwal, Manasi S Shah

**Affiliations:** 1 Internal Medicine, Eastern Virginia Medical School, Norfolk, USA; 2 Endocrinology, Diabetes and Metabolism, Eastern Virginia Medical School, Norfolk, USA

**Keywords:** volume, diuretics, parathyroid hormone, parathyroidectomy, cardiovascular mortality, primary hyperparathyroidism

## Abstract

Primary hyperparathyroidism (PHPT) is an excessive parathyroid hormone (PTH) production disorder, causing increased calcium levels. Commonly, these cases are asymptomatic and detected incidentally on routine labs. These patients are usually conservatively managed and monitored periodically, including bone and kidney health evaluation. Medical management of severe hypercalcemia secondary to PHPT includes IV fluids, cinacalcet, bisphosphonates, and dialysis, while the surgical treatment is parathyroidectomy. Patients suffering from heart failure with reduced ejection fraction (HFrEF) on diuretics and PHPT require a delicate balance of their volume status to prevent exacerbation of either condition. In patients with these two comorbidities on the opposite ends of the volume spectrum, it can lead to challenges in managing these patients. We present a case of a woman with repeated hospitalizations due to poor volume status control.

An 82-year-old female with primary hyperparathyroidism (diagnosed 17 years ago), HFrEF due to non-ischemic cardiomyopathy, sick sinus syndrome with a pacemaker, and persistent atrial fibrillation presented to the emergency department with worsening bilateral lower limb swelling for several months. The remaining review of systems was largely negative. Her home medication regimen included carvedilol, losartan, and furosemide. Vitals were stable, and the physical exam revealed bilateral lower extremity pitting edema. Chest x-ray revealed cardiomegaly with mild pulmonary vascular congestion. Relevant labs were NT pro-BNP at 2190 pg/mL, calcium at 11.2 mg/dL, creatinine at 1.0 mg/dL, PTH at 143 pg/mL, and Vitamin D, 25-hydroxy at 48.6 ng/mL. The echocardiogram showed an ejection fraction (EF) of 39%, grade III diastolic dysfunction, severe pulmonary hypertension, and mitral and tricuspid regurgitation. The patient received IV diuretics and guideline-directed treatment for congestive heart failure exacerbation. She was managed conservatively for her hypercalcemia and advised to maintain hydration at home. Spironolactone and Dapagliflozin were added to her regimen, and the Furosemide dose was increased at discharge.

The patient was re-admitted three weeks later with fatigue and decreased fluid intake. Vitals were stable; however, the physical exam revealed dehydration. Pertinent labs were calcium at 13.4 mg/dL, potassium at 5.7 mmol/L, creatinine at 1.7 mg/dL (baseline 1.0), PTH at 204 pg/mL, and Vitamin D, 25-hydroxy at 54.1 ng/mL. Repeat ECHO showed an ejection fraction (EF) of 15%. She was started on gentle IV fluids to correct the hypercalcemia while preventing volume overload. Hypercalcemia and acute kidney injury improved with hydration. She was put on Cinacalcet 30 mg, and home medications were adjusted for better volume control at discharge.

This case highlights the complications of balancing the volume status with primary hyperparathyroidism and CHF. Worsening HFrEF resulted in a higher diuretic requirement, thereby worsening her hypercalcemia. With emerging data on the correlation between PTH and cardiovascular risks, it is becoming necessary to assess the risks and benefits of conservative management in asymptomatic patients. Current research has also shown that various patient demographics and comorbidities prevent the surgical management of PHPT. Hence, in suitable candidates, parathyroidectomy must be considered early in patients with asymptomatic hyperparathyroidism.

## Introduction

Primary hyperparathyroidism (PHPT) is a condition where excessive parathyroid hormone production results in increased calcium levels, usually due to a parathyroid adenoma. Commonly, these cases are asymptomatic and detected incidentally on routine labs. They can be managed conservatively with sufficient fluid intake and regular calcium, creatinine, and bone density monitoring. Medical management of hypercalcemia due to primary hyperparathyroidism includes intravenous fluids, cinacalcet, bisphosphonates, and even dialysis, while the surgical treatment is parathyroidectomy. Patients suffering from congestive heart failure (CHF) on diuretics and PHPT require a delicate balance of their volume status to prevent exacerbation of either condition. It can be challenging to manage patients with these two comorbidities, which reside on opposite sides of the volume spectrum. Here, we present a case of an elderly woman with repeated hospitalizations due to volume imbalance. 

This article was previously presented as a poster abstract at the EVMS Research Day on October 14, 2022. This article will be presented as a poster abstract at ENDO 2023 on June 15, 2023.

## Case presentation

**Figure 1:**
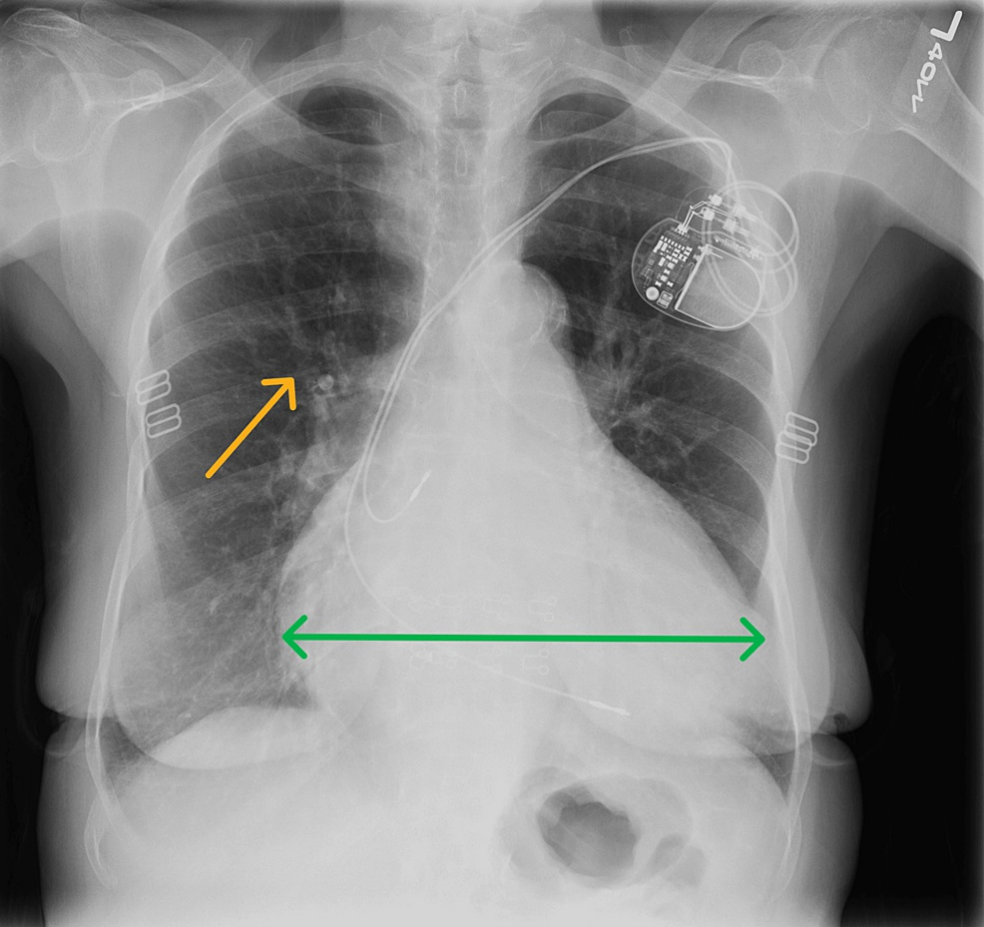
Chest X-Ray Suggestive of Volume Overload Green Arrow- Cardiomegaly; Yellow Arrow- Pulmonary Congestion

An 82-year-old female with HFrEF due to non-ischemic cardiomyopathy, PHPT (diagnosed 17 years ago), sick sinus syndrome with a pacemaker, and persistent atrial fibrillation presented to the emergency room with worsening bilateral lower extremity edema present over the past couple of months. The remaining review of systems was largely negative. Her home medication regimen consisted of carvedilol 12.5 mg twice daily, furosemide 20 mg daily, losartan 50 mg daily, and warfarin. Vital signs were stable, and the physical exam was significant for 2+ bilateral lower extremity pitting edema. Chest x-ray was suggestive of cardiomegaly with mild pulmonary vascular congestion (Figure 1).

Laboratory investigations revealed an NT pro-BNP of 2190 pg/mL, calcium of 11.2 mg/dL, albumin 3.5 g/dL, parathyroid hormone (PTH) of 143 pg/mL, and vitamin D, 25-hydroxy of 48.6 ng/mL (Table 1). The patient received intravenous diuretics and guideline-directed medical therapy (GDMT) for a heart failure exacerbation. A repeat echocardiogram (ECHO) showed an ejection fraction of 39%, grade III diastolic dysfunction, severe biatrial enlargement, severe pulmonary hypertension (pulmonary arterial systolic pressure 74mmHg), and severe mitral regurgitation. A conservative approach was taken for her hypercalcemia. She was additionally advised to maintain hydration at home while maintaining the fluid restriction and resume her Vitamin D supplementation. The following modifications were made to her GDMT: Furosemide 30 mg, Spironolactone 12.5mg, and Dapagliflozin 10 mg daily.

**Table 1:** Lab Value Comparison Between Admissions

Lab	Normal Range	Admission 1	Admission 2
Creatinine	0.8-1.4 mg/dL	1.1	1.7
Potassium	3.5-5.5 mmol/L	4.0	5.7
Calcium	8.4-10.5 mg/dL	11.3	13.4
Ionized Calcium	4.4-5.4 mg/dL	5.6	6.2
Albumin	3.5-4.5 g/dL	3.5	4.4
Phosphorus	2.5-4.5 mg/dL	-	3.4
PTH	15-65 pg/mL	143	204
Vitamin D, 25-Hydroxy	32-100 ng/mL	48.6	54.1
NT-proBNP	<1800 pg/mL (Age corrected)	2190	-

The patient returned to the emergency room three weeks later with symptoms of fatigue, decreased appetite, and decreased fluid intake. She reported she had been compliant with her medications and fluid restriction. Her vital signs were stable, and the physical exam was remarkable for dry oral mucous membranes. Laboratory investigations were significant for calcium 13.4 mg/dL, albumin 4.4 g/dL, potassium 5.7 mEq/L, creatinine 1.7 mg/dL (baseline 1.0), PTH 204 pg/mL, and Vitamin D, 25-hydroxy 54.1 ng/mL (Table 1). An echocardiogram was repeated, now with an ejection fraction of 15%. She was started on gentle intravenous fluids, keeping in mind the worsening congestive heart failure. Her hypercalcemia and acute kidney injury improved with hydration. She was initiated on cinacalcet 30 mg daily due to concern for QTc prolongation on a recent EKG. After a five-day admission, at discharge, the following modifications were made to her GDMT for better volume status control: losartan was discontinued, carvedilol was reduced to 6.25mg twice daily, furosemide was decreased to 20 mg daily, and continued spironolactone and dapagliflozin. 

Unfortunately, the patient returned to the emergency room one week later with similar complaints of dehydration, decreased oral intake, and fatigue. Relevant labs included calcium of 13.3 mg/dL, sodium of 131 mEq/L, and a urinalysis suggestive of a urinary tract infection. The patient received intravenous fluid boluses and oral antibiotics and was discharged home.

## Discussion

PHPT is caused by the abnormal secretion of PTH by one of the parathyroid glands in the form of a parathyroid adenoma or hyperplastic disease. PHPT is most commonly seen in post-menopausal women, with a female-to-male ratio of 3 to 4:1. Its current prevalence is 0.86% in the general US population [1]. Excess PTH causes an increase in bone resorption, calcium absorption in the intestines, and decreased urinary calcium excretion in the kidneys. High plasma calcium levels can present with constipation, altered mental status, nephrolithiasis, arrhythmias, and fractures [1]. In addition, patients with CHF on diuretics with PHPT have a dual causality in worsening their calcium levels. Diuresis and impaired calcium balance result in a hyperosmolar state that can aggravate hypercalcemia.

Diuretics are one of the primary treatment modalities in patients with HFrEF. Loop and thiazide diuretics indirectly modify the calcium metabolism in the kidneys by their action on the various transporters [2]. Loop diuretics act in the thick ascending limb on the Na-K-2Cl cotransporter. Normally, this cotransporter creates a net electro-positivity, generating an electrical gradient resulting in the reabsorption of some of the cations. Loop diuretics, such as furosemide, block the action of this cotransporter and cause an increase in the luminal concentration of sodium chloride, which is excreted out, resulting in natriuresis. In general, loop diuretics cause calciuresis with minimal change to serum calcium concentration [2]. 

Thiazide diuretics act on the Na-Cl cotransporter in the distal tubule by decreasing sodium chloride reabsorption resulting in diuresis. In addition, the overall volume depletion contributes to calcium reabsorption in the distal tubule. Hence, thiazides decrease calciuria, reducing kidney stone formation and creating a positive calcium balance. Thiazides, as a result, can precipitate hypercalcemia in an individual prone to increased levels. A descriptive study found that thiazides were closely associated with hypercalcemia and often unmasked underlying primary hyperparathyroidism [3].

Spironolactone acts on the mineralocorticoid receptors and has a potassium-sparing effect compared to other anti-hypertensives and diuretics. It may also have a calcium-sparing effect [4], leading to decreased urinary calcium excretion. However, spironolactone causes sodium and water loss from its mineralocorticoid receptor antagonist action, leading to a volume deficit. The latest treatment modality for congestive heart failure includes SGLT-2 inhibitors which act on the proximal tubules of the sodium-glucose cotransporter resulting in osmotic diuresis. The increased diuresis can result in volume depletion and precipitation of hypercalcemia in prone individuals [2].

Increasingly, the effect of elevated PTH levels on the cardiovascular system is being recognized. The high concentration of circulating PTH has been known to cause endothelial and arterial dysfunction, creating a connection between elevated PTH levels and cardiovascular mortality [5]. This can be attributed to the presence of PTH receptors on vascular smooth muscles and endothelial cells [6]. In addition, Soares et al. found that PTH elevated above the mean of 55.8 pg/mL was associated with left ventricular hypertrophy in patients > 80 years [7].

Over the years, evidence has shown that the parathyroid hormone itself is a biochemical marker for the severity of heart failure and can predict poorer cardiovascular outcomes. Meng et al. conducted a meta-analysis to determine the correlation between PTH and heart failure. Their statistical analysis of six prospective observational studies with over 25,000 patients revealed that higher PTH levels were associated with a statistically significant increased risk of heart failure (HR 1.75, 95% CI 1.38-2.22) [8]. Similarly, van Ballegooijen et al. established a positive correlation between higher levels of PTH and cardiovascular events [9].

Hagström et al. observed almost a decade ago that pro-atherosclerotic dyslipidemia (a known risk factor for cardiovascular disease) was corrected after parathyroidectomy in post-menopausal women with mild, asymptomatic PHPT [10]. In a more recent study, Axelsson et al. found that untreated PHPT was associated with a greater cardiovascular risk than the matched control participants. Moreover, the risk was reduced significantly for patients who had undergone a parathyroidectomy. This study brings to light the effect of PHPT on cardiovascular health and the benefit of parathyroidectomy [11]. 

There are two main surgical modalities for PHPT, namely minimally invasive parathyroidectomy and bilateral exploration, both with cure rates of 95-99% [12]. Current guidelines from the American Association of Endocrine Surgeons recommend parathyroidectomy [12] for (1) symptomatic PHPT, (2) age < 50 years, (3) calcium > 1mg/dl above the normal range, (4) nephrolithiasis, nephrocalcinosis, or 24-hour urine calcium > 400mg/dL, (5) osteoporosis fragility fracture, and (6) eGFR < 60 mL/min. Even if a patient meets these criteria, the percentage of older adult patients who undergo parathyroidectomy is low (approximately 30%) within the first year of diagnosis [13]. This percentage further declines as patients age, with age being a significant contributing factor [14]. Many factors contribute to the higher percentage of non-surgical management of PHPT in older patients, such as age, comorbidities, and frailty [13]. Even though parathyroidectomy has a greater than 95% cure rate [12,15] and is associated with significant benefits compared to medical management, patients are not given this option due to the above factors, even if they have a high likelihood of survival for the next 10 to 15 years [16].

## Conclusions

This case highlights the challenges of balancing the volume status with primary hyperparathyroidism and congestive heart failure. The use of several heart failure medications and a variable volume status poses a great challenge in adequately managing hypercalcemia in patients with primary hyperparathyroidism. Recent studies highlight that elevated parathyroid hormone levels can influence cardiovascular mortality. Parathyroidectomy has a high cure rate and must be considered early on to decrease cardiovascular events and improve bone strength, renal health, and quality of life in these patients. A multidisciplinary approach involving an endocrinologist, primary care physician, and surgeon must be taken when dealing with older adults who may be potential candidates for a parathyroidectomy. Keeping these new findings in mind, primary care physicians and endocrinologists must consider early diagnosis and surgical management of even asymptomatic primary hyperparathyroidism patients to prevent cardiovascular complications later. 
